# Spontaneous conception and pregnancy outcomes after multi‐agent chemotherapy and high‐dose radiotherapy for pelvic Ewing sarcoma: Case report and literature review

**DOI:** 10.1002/ijgo.70029

**Published:** 2025-02-21

**Authors:** Valentina Tosto, Federico Prefumo, Carolina Scala, Ramona Tallone, Alberto Garaventa, Maurizio Podestà, Ilaria Maffeo

**Affiliations:** ^1^ Obstetrics and Gynecology Unit IRCCS Istituto Giannina Gaslini Genoa Italy; ^2^ D.O.P.O. Clinic, Department of Pediatric Hematology and Oncology IRCCS Istituto Giannina Gaslini Genoa Italy; ^3^ Oncology Unit IRCCS Istituto Giannina Gaslini Genoa Italy; ^4^ Academic Unit of Obstetrics and Gynecology IRCCS Ospedale San Martino Genoa Italy

**Keywords:** delivery, management, obstetric outcomes, pelvic Ewing sarcoma, pregnancy

## Abstract

Ewing sarcoma is an aggressive, rare bone and soft tissue malignancy, often affecting the long bones and pelvis. A woman was diagnosed with Ewing sarcoma of the left pelvis at 18 years of age. She underwent left paravesical lesion resection with ipsilateral ureteral reimplantation and received combined multi‐agent chemotherapy and high‐dose pelvic radiotherapy. A gonadotropin‐releasing hormone analog was administered to preserve ovarian function. The patient received oral contraceptives as hormone replacement therapy due to secondary amenorrhea. She spontaneously conceived after the withdrawal of oral contraceptives at 24 years. The pregnancy was complicated by early third‐trimester impaired fetal growth and preterm premature rupture of membranes at 32 weeks of pregnancy. Spontaneous preterm labor started and the patient delivered vaginally at 34 weeks, without any complications. Including our report, only 10 cases of pregnancy after pelvic Ewing sarcoma are reported in the literature. Pregnancy and delivery can be achieved after combined pelvic treatment for Ewing sarcoma, taking into account possible obstetric risks and complications. Complex bone surgery is associated with cesarean delivery, but previous high‐dose pelvic irradiation does not seem to affect mode of delivery. Our report provides further information on perinatal management, given the cases previously described.

## 1 INTRODUCTION

Ewing sarcoma is an aggressive, rare bone and soft tissue malignancy, often affecting the long bones and pelvis. It is the second most common primary cancer in children, adolescents, and young adults. Around 80% of cases are found in patients under 18 years of age and less than 1% of cases are found in adults older than 40 years. It has an estimated incidence in 0.3/100 000 per year in white Caucasians younger than 25 years old.^1^, ^2^ Ewing sarcoma may have a prolonged diagnosis interval because of non‐specific presenting complaints. In particular, in the younger population, Ewing sarcoma may be first misdiagnosed as a traumatic injury, tendinitis, septic arthritis, or others signs/symptoms depending on the primary tumor site (including rarely also soft tissues of abdomen and chest).^1^

Local management options for pelvic Ewing sarcoma include surgery, (neo)adjuvant chemotherapy and radiation therapy, variably combined.^3^ Cumulative data on 5‐ and 10‐year survival rates are reported to be around 65%–80% for non‐metastatic disease.^3^, ^4^ Pregnancy following treatment for Ewing sarcoma of the pelvis is very rare and few cases are reported.^5^, ^6^, ^7^, ^8^, ^9^, ^10^ Here, we present a case of pregnancy and related perinatal management in a patient who underwent combined intensive treatments for pelvic Ewing sarcoma. A literature review was also performed.

## 2 CASE REPORT

A woman was diagnosed with Ewing sarcoma of the left pelvis at 18 years of age. She underwent left paravesical lesion resection with ipsilateral ureteral reimplantation; the surgery was complicated by a vascular accident that required emergency open surgery with a longitudinal umbilico‐pubic incision. Subsequently, she received six courses of multi‐agent chemotherapy with vincristine, cyclophosphamide, ifosfamide, etoposide, dactinomycin, and doxorubicin. Pelvic radiotherapy with a total dose of 54 Gy and without shielding was also performed. A few months later, she underwent a second surgery for resection of hepatic and pararenal metastases. Subsequent checks of the primary site and other possible metastatic disease were achieved, and the clinical outcome was good.

The woman received monthly gonadotropin‐releasing hormone analog administration to preserve ovarian function during adjuvant therapy. She exhibited secondary amenorrhea after completion of treatment and subsequently received oral contraceptives as hormone replacement therapy. Assessment of ovarian reserve was performed with serum anti‐Müllerian hormone (AMH), follicle‐stimulating hormone (FSH), and luteinizing hormone (LH) measurements and ultrasound antral follicle count was evaluated. At 20 years of age, AMH concentration was 0.010 ng/mL, FSH was 137 mUI/mL, and LH was 56 mUI/mL.

The woman was in a relationship at the age of 23 years and discontinued oral contraceptives. A normal menstrual cycle was restored, and she became spontaneously pregnant a few months later. The first and second trimesters of pregnancy were uneventful. Routine fetal ultrasound showed a normal growth pattern until the early third trimester (30 weeks), when a further scan demonstrated estimated fetal weight at the 5th centile, with normal umbilical artery Doppler and normal amniotic fluid volume.

Spontaneous preterm premature rupture of membranes occurred at 32 weeks of pregnancy, together with uterine contractions. Transvaginal ultrasound showed a cervical length of 16 mm. Estimated fetal weight was at the 4th centile, with normal Doppler findings. Computerized cardiotocography was normal.

A full course of betamethasone for respiratory distress syndrome prophylaxis, a 48‐h cycle of intravenous atosiban for tocolysis, and 7 days of intravenous antibiotics were administered. Conservative management with regular checks for infection with clinical and laboratory parameters, and maternal‐fetal surveillance ensued during the next 2 weeks. Spontaneous preterm premature labor started at 34 weeks of pregnancy, and a healthy female baby weighing 1830 g was delivered vaginally, with Apgar scores of 8 at 1 min and 9 at 5 min.

Postpartum and puerperium were uneventful. The mother was discharged 7 days after delivery, but the neonate remained hospitalized for 19 days because of prematurity, without major complications.

The histologic examination found a low‐weight placenta for the gestational age and associated signs of villitis and chronic intervillitis consistent with a state of preterm premature rupture of membranes.

No ethics committee approval was necessary for this study according to local regulations. Written informed consent was obtained from the patient.

## 3 DISCUSSION

We describe a case of spontaneous pregnancy and delivery in a patient who underwent intensive combined treatments for pelvic Ewing sarcoma. The advances of Ewing sarcoma therapeutic strategies resulting from multidisciplinary management (surgeons, oncologists, radiotherapists, radiologists) improved oncologic outcomes. The current 5‐ to 10‐year overall survival for patients with localized disease is 65%–80%.^3^, ^4^

Based on histologic type and anatomical tumor localization, therapeutic strategies can be combined, with possible heterogeneous extra‐oncologic results, including impact on fertility, post‐irradiation sequelae, and motor disability related to surgical resection/reconstruction techniques.

In this regard, Ewing sarcoma of the pelvis was initially treated with radical hemipelvectomy together with amputation of the limb of the affected side. More recently, limb‐sparing surgery showed similar oncologic results as well as a significant improvement in the quality of life for the patients, becoming the treatment of choice for the possibility of preserving the ability of patients to walk and reducing the psychological impact. The type of surgical procedure should be tailored according to the anatomical location of the lesion, extent of resection, patient's functional demands, and surgeon preference.^11^

Only nine cases of successful pregnancy after pelvic Ewing sarcoma treatments have been reported in the literature.^5^, ^6^, ^7^, ^8^, ^9^, ^10^, ^12^ Previous cases and the present one are summarized in Table 1. The woman described by Hockert and Velickovic^10^ also suffered from an early miscarriage at 8 weeks.

**TABLE 1:** Reported pregnancies after treatment of pelvic Ewing sarcoma.

Reference	Tumor site	Age at diagnosis, years	Fertility preservation	Treatment	Age at conception, years	Mode of conception	Pregnancy course	Timing and mode of delivery	Neonatal weight, g
Chihara et al. 2003^6^	Left pelvic bone	11	NA	Surgery and chemotherapy	22	Spontaneous	Maternal walking difficult due to pelvic distortion	Elective CS at 37 weeks (indication pelvic surgery)	Male, 2345 g
Bath et al. 2004^7^	Left superior pubic ramus	14	Ovarian tissue cryopreservation	Chemotherapy and radiotherapy	20	Spontaneous	Uneventful course	Elective CS at 38 weeks (indication pelvic radiotherapy)	Male, 2940 g
Kakogawa et al. 2015^8^	Sacrum	16	GnRH, uterine shielding, laparoscopic ovarian transposition	Surgery, chemotherapy, radiotherapy	27	Spontaneous	Uneventful course	Elective CS at 37 weeks (indication pelvic radiotherapy)	Female, 2326 g
Rodriguez‐Wallberg et al. 2015^9^	Sacrum	23	Ovarian tissue cryopreservation	Chemotherapy and radiotherapy	37	Assisted (ovarian transplantation plus IVF)	Uneventful course	Elective CS at 38 weeks (indication NA)	Female, 2970 g
Hockert and Velickovic 2018^10^	Sacrum	18	None	Surgery, chemotherapy, radiotherapy	29	Spontaneous	Uneventful course	Elective CS at 37 weeks (indication pelvic surgery)	Male, 3460 g
Ciganda et al. 2020^15^	Left iliac bone	13	None	Surgery, chemotherapy, radiotherapy	29	Assisted (IVF)	Spontaneous uterine rupture	Hysterectomy with intrauterine fetus at 23^+4^ weeks	Stillbirth
Gutkin et al. 2020^12^	Left iliac bone and extra‐pelvic soft tissue	16	None	Chemotherapy, radiotherapy	28	Spontaneous (two conceptions)	Uneventful course	Term vaginal delivery, no complications	Female, 3175 g
					32	Spontaneous	Uneventful course	Term vaginal delivery, no complications	Male, 2812 g
Kakogawa et al. 2021^5^	Left pelvic bone	17	None	Surgery, chemotherapy, radiotherapy	25	Spontaneous	Maternal walking difficult due to pelvic distortion	Elective CS at 37 weeks (indication pelvic surgery)	Female, 2464 g
Current case 2025	Left paravesical lesion	18	GnRh analog	Surgery, chemotherapy, radiotherapy	24	Spontaneous	PPROM, FGR, threatened preterm birth	Spontaneous preterm delivery at 34 weeks, no complications	Female, 1830 g

Abbreviations: CS, cesarean section; GnRH, gonadotropin‐releasing hormone; FGR, fetal growth restriction; IVF, in vitro fertilization; NA, not available; PPROM, preterm premature rupture of membranes.

All cases underwent chemotherapy and/or radiotherapy. Two cases did not require surgery. The impact of treatment on fertility is a significant concern for survivors of childhood and adolescent cancer, because of the higher risk of premature ovarian failure as a result of the potentially gonadotoxic treatment (massive follicle loss due to direct toxicity on the oocyte and vascular damage).

There are now options for fertility preservation, even if limited, and accurate reproductive counseling should always be offered and well documented. In two cases ovarian tissue cryopreservation was performed in young women with pelvic Ewing sarcoma^7^, ^9^ and one of them conceived by in vitro fertilization following ovarian transplantation.^9^ However, an exact and absolute prediction of reproductive performance/fertility chance is not possible, as six out of seven cases (including the current one) were successful in conceiving spontaneously.

Not only ovarian function, but also uterine function may be negatively affected by oncologic treatments The need for high‐dose pelvic radiation, without the possibility of shielding, exposes the patient to considerably higher risks of compromising future fertility and obstetrics outcomes. Timing (exposure before or after puberty) and dose (doses greater than 10 Gy after puberty or lower doses of 1–2.5 Gy before puberty are associated with an increased risk of stillbirth and neonatal death) of uterine exposure to radiation appear to be critical determinants of the magnitude of damage and poor obstetric outcomes.^13^, ^14^

Infertility and pregnancy complications reported in patients who received pelvic radiation include premature ovarian failure, miscarriage, fetal growth restriction, preterm birth, pre‐eclampsia, abnormal placentation, delivery of small‐for‐gestational‐age infants, fetal malposition, and perinatal death.^13^ Radiation‐induced damage in the endometrium with vascular alterations may predispose to impaired decidualization, placental development, and attachment, if pregnancy can be achieved. Furthermore, uterine elasticity and volume can be reduced by radiation‐induced myometrial damage, which can lead to preterm labor and delivery. The current case showed these possible consequences: the patient had a premature preterm rupture of membranes and a preterm delivery, and the fetus experienced impaired intrauterine growth. As evidence of the possible uterine risks connected to pelvic irradiation, Ciganda et al.^15^ described a case of acute abdominal pain and hemoperitoneum following spontaneous uterine rupture at 23^+4^ weeks of gestation in a woman with previous high‐dose pelvic radiotherapy due to Ewing sarcoma. This woman received an emergency hysterectomy with intrauterine fetus. Regarding the mode of delivery, there is no evidence to support a cesarean section over a vaginal delivery. Review of the literature showed that seven out of ten cases delivered through cesarean section, because of anatomical and functional sequelae of surgery and radiotherapy (severe pelvic distortion with difficult ambulation). Three women, including our case, successfully gave birth vaginally.^12^ Based on the data available regarding the mode of delivery, we suggest that: (1) not having undergone a complex bone surgery may represent a crucial advantage for opting for a trial of labor and vaginal delivery; (2) having received high‐dose pelvic irradiation is not a certain cause of myometrial dysfunction, which contraindicates vaginal birth. To date, the literature reports only one case of a patient with history of pelvic Ewing sarcoma who had two pregnancies, both with spontaneous conception, a regular antenatal course and vaginal birth without complications.^12^

In conclusion, we should expect an increasing number of surviving women treated for pelvic Ewing sarcoma in the future. Pregnancy and delivery can be spontaneously and successfully achieved. Multidisciplinary management of women with pelvic Ewing sarcoma is suggested for the need to consider a number of issues, including accurate reproductive counseling regarding future fertility and obstetric risks (if pregnancy is desired and achieved). Our report aims to provide a further contribution on perinatal management, given the rarity of cases previously described.

## AUTHOR CONTRIBUTIONS

VT, IM, and FP conceived and designed this study; VT, RT, and MP contributed substantially to the acquisition of the data; FP, IM, CS, AG and VT contributed to the interpretation of the results; and VT and IM drafted the paper. All authors revised and approved the final version of the manuscript.

## CONFLICT OF INTEREST STATEMENT

The authors have no conflicts of interest.

## Data Availability

Data available on request due to privacy/ethical restrictions.
